# Purification and Characterization of Melanogenic Enzyme Tyrosinase from Button Mushroom

**DOI:** 10.1155/2014/120739

**Published:** 2014-08-14

**Authors:** Kamal Uddin Zaidi, Ayesha S. Ali, Sharique A. Ali

**Affiliations:** ^1^Molecular Biotechnology Laboratory, Centre for Scientific Research & Development, People's University, Bhopal 462010, India; ^2^Department of Biotechnology, Saifia College of Science, Bhopal 462001, India

## Abstract

Melanogenesis is a biosynthetic pathway for the formation of the pigment melanin in human skin. A key enzyme, tyrosinase, catalyzes the first and only rate-limiting steps in melanogenesis. Since the discovery of its melanogenic properties, tyrosinase has been in prime focus and microbial sources of the enzyme are sought. *Agaricus bisporus* widely known as the common edible mushroom, it's taking place in high amounts of proteins, enzyme, carbohydrates, fibers, and low fat contents are frequently cited in the literature in relation to their nutritional value. In the present study tyrosinase from *Agaricus bisporus* was purified by ammonium sulphate precipitation, dialysis followed by gel filtration chromatography on Sephadex G-100, and ion exchange chromatography on DEAE-Cellulose; the enzyme was purified, 16.36-fold to give 26.6% yield on total activity in the crude extract and final specific activity of 52.19 U/mg. The SDS-PAGE electrophoresis showed a migrating protein band molecular weight of 95 kDa. The purified tyrosinase was optimized and the results revealed that the optimum values are pH 7.0 and temperature 35°C. The highest activity was reported towards its natural substrate, L-DOPA, with an apparent Km value of 0.933 mM. This indicated that tyrosinase purified from *Agaricus bisporus* is a potential source for medical applications.

## 1. Introduction

Tyrosinase (E.C. 1.14.18.1) is a ubiquitous enzyme involved in pigmentation. It catalyzes hydroxylation of monophenols (cresolase activity) and oxidation of diphenols (catecholase activity) in the presence of molecular oxygen. The conversion of phenols to* o*-diphenols by tyrosinase is a potentially attractive catalytic ability and thus tyrosinase has attracted a lot of attention with respect to its biotechnological application as the catechol products are useful as drugs or drug synthons, for example, L-DOPA [1]. It also plays an important role in the formation of melanin pigment during melanogenesis in melanocytes which are located at the epidermal junction and it is present in these cells that originate from the embryonic neural crest and is responsible for the synthesis of melanin. [2] It was first characterized in mammals for its role in the development of melanomas and for implication in pigmentation troubles such as albinism and vitiligo [3]. The physiological role of tyrosinases is related to melanin biosynthesis and has been extracted from different sources such as fungi, fruits, and mammalian melanoma tumors [4, 5]. In fungi, melanins are involved in defence mechanisms against stress factors such as UV or gamma radiation, free radicals, dehydration, and extreme temperatures [6, 7]. The stability of fungal spores also benefits from the protective role of melanin [8]. In addition, tyrosinases are associated with wound healing, with the immune response in plants [9, 10]. In humans, tyrosinase is involved in the pigmentation in melanocytes [11–13], as a marker in melanoma patients [14] and as a target for the activation of prodrugs [15]. In the present work, as an initial step in evaluating the pharmaceutical potential of the tyrosinase of mushroom* Agaricus bisporus*, we undertook a preliminary characterization of the enzyme. The characterized tyrosinase showed very high similarities compared to human tyrosinase; this indicated that purified and characterized mushroom can be a prosperous source of tyrosinase for therapeutic use in melanogenesis.

## 2. Materials and Methods

### 2.1. Preparation of Tyrosinase

Extraction of mushroom tyrosinase was performed by the method of Kamahldin et al. [16], with few modifications. The sliced mushrooms were homogenized by waring blender. Enzyme extraction was prepared with 500 mL of cold 100 mM phosphate buffer (pH 5.8) for 300 g of mushroom. The homogenate was centrifuged at 5000 rpm for 30 min and supernatant was collected. The sediments were mixed with cold phosphate buffer and were allowed to stand in cold condition with occasional shaking. Then the sediment containing buffer was subjected to centrifugation once again to collect supernatant. The supernatant was used as a source of enzyme.

### 2.2. Purification of the Enzyme from the Crude Extract

The purification of tyrosinase was performed by the method of Kamahldin et al. [16], with minor modification. Crude enzyme extract purified by salt precipitation, dialysis, gel filtration, ion exchange chromatography, and so forth has been employed in series so as to obtain the enzyme in its purest form. The pure enzyme thus produced can be used for the further analysis.

### 2.3. Ammonium Sulphate Precipitation and Dialysis

Ammonium sulphate precipitation was done in an ice bath using the finely grounded ammonium sulfate. The powder was weighed and added slowly to the extract by constant stirring to ensure complete solubility, and the solution was centrifuged at 5000 rpm for 30 min at 4°C. Different precipitation steps were carried out for tyrosinase enzyme precipitation (45–80%) and precipitates were collected. The precipitate was dialyzed against 100 mM potassium phosphate buffer (pH 7.0) for 24 h by changing the buffer thrice. The dialyzed fraction was used for tyrosinase activity and protein content.

### 2.4. Assay of Tyrosinase Activity

The tyrosinase activity assay was performed as reported by Sung and Cho [17] spectrophotometrically, measuring conversion of L-DOPA to red colored oxidation product dopachrome. The initial rate of reaction is proportional to concentration of the enzyme. An aliquot containing tyrosinase was incubated for 5 min at 35°C at time zero, 1 mL of L-DOPA solution (4 mg/mL) for measured at 475 nm. After incubation for additional 5 min, the mixture was shaken again and a second reading was determined and was measured for 3 minutes. The change in absorbance was proportional to enzyme concentration. One unit of enzyme corresponded to the amount which catalyzed the transformation of 1 *μ*mol of substrate to product per min under the above conditions and produced 1.35 changes in absorbance. Specific activity was expressed as enzyme unit per milligram of protein. The protein content of the enzyme was determined by the method of Lowry [18], with bovine serum albumin as standard.

### 2.5. Sephadex G-100 Gel Filtration

The dialyzed ammonium sulfate fraction was applied to a Sephadex G-100 column that was preequilibrated with a 100 mM phosphate buffer of pH 7.0. The protein elution was done with the same buffer at a flow rate of 5 mL/min. The fractions were collected at 4°C. It was assayed for protein at 280 nm as well as for enzyme activity. The active fractions were pooled, dialyzed against the 100 mM phosphate buffer of pH 7.0, and concentrated.

### 2.6. DEAE-Cellulose Column Chromatography

Dialyzed enzyme preparation obtained after ammonium sulphate precipitation and Sephadex G-100 column was subjected to ion exchange chromatography using DEAE-Cellulose column (20 × 1 cm). The dialyzed enzyme preparation was loaded on DEAE-Cellulose column which was preequilibrated with potassium phosphate buffer (100 mM, pH 7.0). The column was washed first with equilibrated buffer and then bound proteins were eluted using linear gradient of 0–100 mM NaCl and 0–100 mM potassium phosphate buffer at a flow rate of 1 mL per min. The fractions (2.5 mL each) were collected and assayed for tyrosinase activity and those showing high activity were pooled and used for SDS-PAGE analysis.

### 2.7. Sodium Dodecyl Sulfate-Polyacrylamide Gel (SDS-PAGE) Electrophoresis of Purified Tyrosinase

SDS-PAGE was performed using a 12% separating gel and 4% stacking gel. The samples were heated for 5 min at 100°C in capped vials with 1% (w/v) SDS in the presence of *β*-mercaptoethanol. Electrophoresis was performed at a 125 V for 4 h in Tris-HCl buffer of pH 8.3. After electrophoresis, proteins in the separating gel were made visible by staining with Coomassie Brilliant Blue R-250. The standards used to make a plot of log molecular weight versus mobility of the protein band were lysozyme (20 kDa), myoglobin (26 kDa), carbonic anhydrase (38 kDa), ovalbumin (46 kDa), glutamate (62 kDa), bovine serum albumin (91 kDa), *β*-galactosidase (120 kDa), and myosin (200 kDa).

### 2.8. Effect of pH and Temperature on Enzyme Activity

The activity of tyrosinase was evaluated at different pH values in the range between pH 3 and 10 under assay conditions and the amount of dopachrome was determined. Buffers used were citrate phosphate (pH 3.0–5.0), potassium phosphate (pH 6.0-7.0), Tris-HCl (pH 8.0-9.0), and glycine-NaOH (pH 9.0-10). Optimum temperature for enzyme activity was determined by incubating the standard reaction mixture at temperatures ranging from 35 to 65°C.

### 2.9. Kinetic Analysis

The enzyme kinetics as measured by the Michaelis constant (Km) is defined as the substrate concentration at half the maximum velocity, the rate of enzymatic reactions, by relating reaction rate to the concentration of a substrate. The Michaelis constant (Km) value of the purified enzyme was estimated in a range of tyrosinase concentrations. The apparent Km value of purified tyrosinase was calculated from the Lineweaver-Burk plots relating 1/V to 1/[S].

## 3. Results and Discussion

### 3.1. Partial Purification of Tyrosinase

Mushroom contains a considerable amount of various phenolic compounds, which are readily oxidized during the homogenizing process. Upon oxidation and successive polymerization of the phenolic content of the mushroom extract, macromolecules of melanins are formed. The purification of tyrosinase from mushroom is moderately more difficult due to the smaller amount of tissue available per fruiting body of mushroom and greater amount of melanin in these tissues. The accompanying melanins which are usually built up during preparation of the homogenized extract could be extensively removed from the protein mixture using ion exchange material. Initially, we tried adsorbent anion exchangers, as well as precipitation methods ammonium sulfate precipitation to remove colored material or to concentrate tyrosinase. None of these methods were successful without causing a decrease in the recovery of enzyme activity or not being able to remove a substantial amount of colored material.

**Figure 1:**
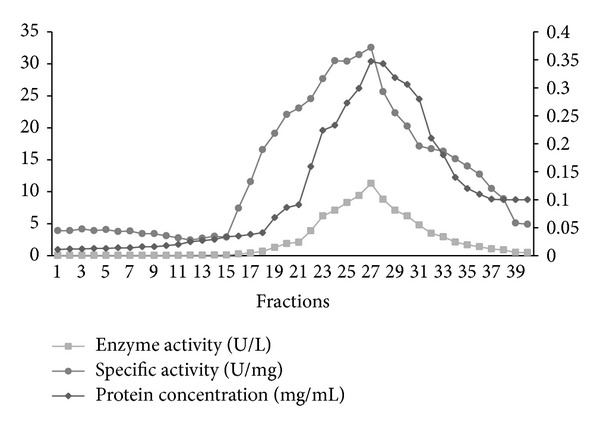
Elution profile on DEAE-Cellulose column chromatography. An aliquot of each fraction was assayed for protein content and tyrosinase activity. A linear gradient of NaCl was 0–100 mM.

**Figure 2:**
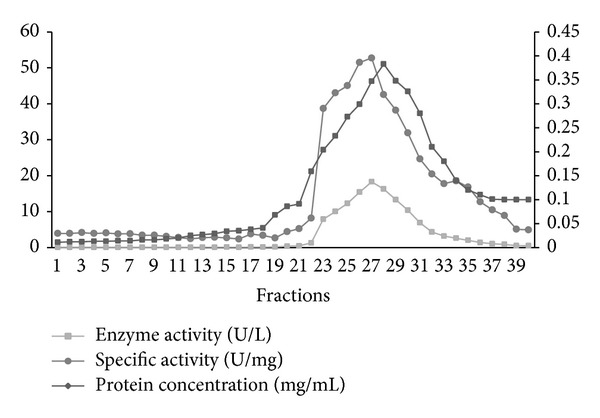
Elution profile on DEAE-Cellulose column chromatography. An aliquot of each fraction was assayed for protein content and tyrosinase activity. A linear gradient of potassium phosphate buffer was 0–100 mM.

Briefly, crude extracts were prepared by homogenization in 100 mM potassium phosphate buffer (pH 5.8) containing 1 mM EDTA. After centrifugation, the supernatant was applied to ammonium sulfate precipitation. Partial purification of tyrosinase using ammonium sulfate precipitation showed that the best fraction was 70% with respect to the crude enzyme and other fractions. It gave the maximum values of total activity, specific activity, and yield of the tyrosinase enzyme which reached 11.09 U/mg, 83.5%, respectively. The -fold purification of the purified enzyme was 3.47 when 70% ammonium sulfate was used. The previous findings were identical to that reported by Lee et al. [19], who found that 70% ammonium sulfate was the best fraction which gave the highest yield of tyrosinase activity from* Solanum melongena.* The dialyzed ammonium sulfate precipitate that was applied to Sephadex G-100 gel filtration column chromatography showed major peaks of tyrosinase activity which were observed in active fractions and resulted in 9.22-fold purification with a final specific activity of 29.42 U/mg. The overall recovery of the purification was 35.7% (Table 1). This active fraction was applied to a DEAE-Cellulose column and eluted with a stepwise-increasing NaCl gradient. The enzyme was eluted at 0–100 mM NaCl (Figure 1). The eluted active fractions rechromatographed on the same column with a linear gradient of potassium phosphate buffer (0–100 mM) were passed through the column (Figure 2). This two-step purification scheme, ion exchange chromatography, resulted in a partially purified tyrosinase preparation, obtained by pooling fractions 25, 26, 27, and 28 and the enzyme was purified by about 16.36-fold purification with a final specific activity of 52.19 U/mg. The overall recovery of the purification was 26.6%. Horowitz et al. [20] reported that tyrosinase that is produced in the fruiting body can be recuperated and purified by homogenizing in a blender and then passed through a French press followed by acetone or ammonium sulfate precipitation [21]. The resuspended precipitate was further purified by one or more chromatography columns. The most commonly used columns are hydroxylapatite [22], DEAE-Cellulose [23] or DEAE Sepharose [24], various other immunoaffinity resins [25], and Sephadex size exclusion gel [21].

**Figure 3:**
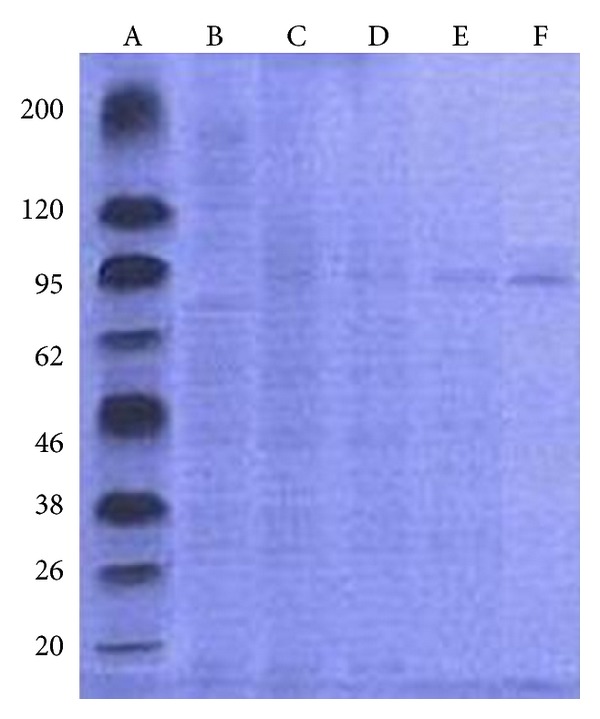
Polyacrylamide gel electrophoresis of tyrosinase from* A. bisporus*, lane A, standard protein of different molecular weight; lane B, crude extract; lane C, ammonium sulfate fraction; lane D, dialysis; lane E, Sephadex G-100 gel filtration fraction; and lane F, DEAE-Cellulose fraction of tyrosinase ~95 kDa.

SDS-PAGE of the enzyme preparation from different purification steps showed that the resolved electrophoretic bands were progressively improved from the crude extract to the final step of the DEAE-Cellulose column. It revealed only a single distinctive protein band for the pure preparation of tyrosinase with an apparent molecular weight of 95 kDa (Figure 3). In this respect, tyrosinase purified from* Aspergillus oryzae*,* Trichoderma reesei*, and* Aspergillus nidulans* was with smaller molecular weight in the range of 67, 43.2, and 50.48 kDa [26–28]. Kanda et al. [29] obtained two activity peaks after ion exchange chromatography of an extract from* Lentinula edodes*. When the fractions corresponding to each peak were analyzed by partially denaturing SDS-PAGE, both had three bands that showed tyrosinase activity. Fully denaturing SDS-PAGE of the same fractions gave bands at 15, 49, and 54 kDa for one fraction and 15, 50, and 55 kDa for the other purified tyrosinase from* Lentinula edodes* [29] and exhibited a molecular weight of 105 kDa.

### 3.2. Properties of the Partially Purified Enzyme

**Figure 4:**
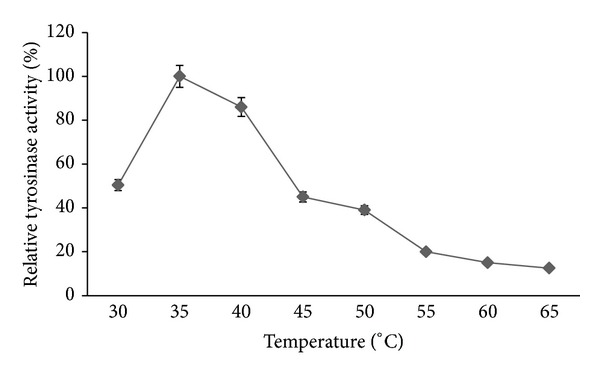
Effect of temperature on the tyrosinase activity of the crude extract prepared from* A. bisporus*. Data were obtained as mean value of optical density. Assays were done in potassium phosphate buffer (100 mM, pH = 7.0). The optimum activity of the sample incubated at 35°C was taken as 100%.

The purified tyrosinase was active at a wide range of temperature from 30°C to 65°C with an optimum at 35°C (Figure 4), and about 35% of tyrosinase activity was still present at 55°C, but it lost its activity at 65°C. Our results were in agreement with a previous study which reported that the optimum temperature for tyrosinase activity obtained from* Streptomyces *sp. was 35°C. Tyrosinase from* Pseudomonas putida* and* Trichoderma reesei* showed maximum activity at 30°C [27, 30], and maximum activity of tyrosinase purified from* Bacillus megaterium* and* Lentinula boryana* was at 40°C [31, 32].

**Figure 5:**
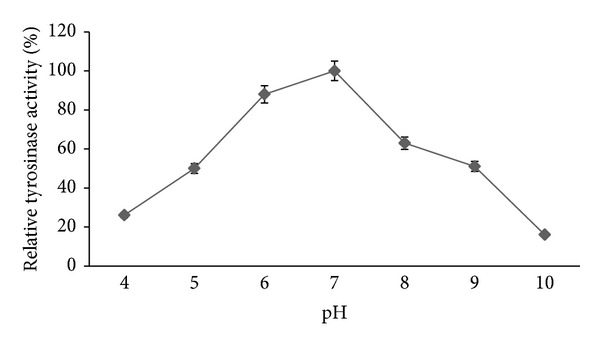
Effect of pH on the tyrosinase activity of the crude extract of* A. bisporus*. Data were obtained as mean value of optical density. Assays were done at 35°C and the activity of the sample was incubated on 100 mM acetate buffer at 4.0-5.0 pH, 100 mM phosphate buffer at 6.0–8.0 pH, and 100 mM Tris-HCl buffer at 9.0-10 pH. The optimum activity of the sample at pH 6.0 was taken as 100%.

Tyrosinases with various physicochemical features have been reported from various organisms. These enzymes generally have a pH optimum in the neutral or slightly acidic range. The tyrosinase from* T. reesei* and* I. batatas* has a basic pH optimum of 9 and 8, respectively [27, 33]. Results (Figure 5) revealed that pH 7.0 was the optimal pH for tyrosinase from* A. bisporus* using phosphate buffer. These results coincide with that of Liu et al. [31] who reported that the maximal tyrosinase activity of* Bacillus megaterium* was 7.0, and the optimal L-tyrosinase activity extracted from* Trichoderma reesei* was 9.0 [27]. The pH-dependent changes in the kinetic properties of the mushroom tyrosinase are similar to the pH-dependent changes in the kinetic properties of tyrosinase from B-16 murine melanoma and human skin and thus appear to be a general property of tyrosinase from diverse sources. Our results also demonstrated that tyrosinase retained about 65 % of its activity after storing at pH 7.0 for 24 h. This means that tyrosinase of* A. bisporus* had higher pH stability over a wide range of pH values.

**Figure 6:**
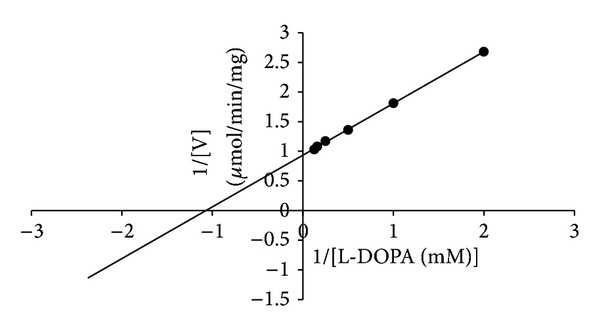
Lineweaver-Burk plot of* A. bisporus *tyrosinase. Data were obtained as mean value of 1/[V], inverse of the increase of optical density at 475 nm per min (OD475), of three independent tests with different concentrations of L-DOPA as a substrate.

The Km value of tyrosinase was found to be 0.933 mM shown in (Figure 6). This result indicates the high affinity of tyrosinase towards its substrate, which might relate to its degree of effectiveness against melanogenesis. Higher Km values 0.9 and 0.85 mM for tyrosinase from* Pycnoporus sanguineus* and* Lentinula edodes*, respectively, have been reported [24, 29]. On The other hand, a lower Km value (0.075 mM) was obtained for tyrosinase from* Bacillus megaterium* [31].

## 4. Conclusions

In the increasingly ageing Western population with significant cumulative sun exposure, the control of hyperpigmentation and dyspigmentation is a significant clinical and cosmetic challenge. Factors like UVR exposure and skin inflammation have been implicated in hyperpigmentary conditions such as melasma and solar lentigines. As dyspigmentation is viewed as a prominent marker of facial skin ageing, novel, safe, and effective agents that modulate pigmentation are being sought. A wide range of agents have been described to treat hyper- and dyspigmentation with different mechanism of action. The key enzyme that is responsible for melanin production is tyrosinase; those modulating strategies should ideally address multiple aspects of melanocyte biology in human skin cell, that is, melanogenesis (induced tyrosinase protein expression using cellular tyrosine) melanin transfer to recipient keratinocytes. From the present study, as an initial step in evaluating the pharmaceutical potential of the tyrosinase of mushroom, we undertook a preliminary characterization of the enzyme. The characterized tyrosinase showed very high similarities compared to human tyrosinase. This indicated that purified and characterized mushroom tyrosinase can be a prosperous source of tyrosinase for therapeutic use in melanogenesis.

## Figures and Tables

**Table 1:** Purification of tyrosinase from mushroom *A. bisporus*.

Fractions	Volume (mL)	Total protein (mg)	Activity (units)	Total activity U	Specific activity U/mg	-fold purification	Yield (%)
Crude extract	400	321.4	2.56	1025	3.189	1	100
Ammonium sulphate precipitate	120	77.13	7.13	856	11.09	3.47	83.5
Dialysis	60	33.63	8.25	495	14.71	4.61	48.2
Gel filtration G100 column	30	12.44	12.2	366	29.42	9.22	35.7
DEAE-Cellulose column	15	5.23	18.2	273	52.19	16.36	26.6

DEAE: diethylaminoethyl.
